# Circulating Prolidase Activity in Patients with Myocardial Infarction

**DOI:** 10.3389/fcvm.2017.00050

**Published:** 2017-07-31

**Authors:** Adnan Sultan, Yuting Zheng, Patrick J. Trainor, Yong Siow, Alok R. Amraotkar, Bradford G. Hill, Andrew P. DeFilippis

**Affiliations:** ^1^Department of Medicine, Division of Cardiovascular Medicine, University of Louisville, Louisville, KY, United States; ^2^Department of Medicine, Division of Cardiovascular Medicine, and Bioinformatics, University of Louisville, Louisville, KY, United States; ^3^KentuckyOne, Jewish Hospital, Johns Hopkins University, Baltimore, MD, United States

**Keywords:** prolidase, atherothrombosis, acute myocardial infarction, diabetes, coronary artery disease

## Abstract

**Background:**

Collagen is a major determinant of atherosclerotic plaque stability. Thus, identification of differences in enzymes that regulate collagen integrity could be useful for predicting susceptibility to atherothrombosis or for diagnosing plaque rupture. In this study, we sought to determine whether prolidase, the rate-limiting enzyme of collagen turnover, differs in human subjects with acute myocardial infarction (MI) versus those with stable coronary artery disease (CAD).

**Methods:**

We measured serum prolidase activity in 15 patients with stable CAD and 49 patients with acute MI, of which a subset had clearly defined thrombotic MI (*n* = 22) or non-thrombotic MI (*n* = 12). Prolidase activity was compared across study time points (at cardiac catheterization, T0; 6 h after presentation, T6; and at a quiescent follow-up, Tf/u) in acute MI and stable CAD subjects. We performed subgroup analyses to evaluate prolidase activity in subjects presenting with acute thrombotic versus non-thrombotic MI.

**Results:**

Although prolidase activity was lower at T0 and T6 versus the quiescent phase in acute MI and stable CAD subjects (*p* < 0.0001), it was not significantly different between acute MI and stable CAD subjects at any time point (T0, T6, and Tf/u) or between thrombotic and non-thrombotic MI groups. Preliminary data from stratified analyses of a small number of diabetic subjects (*n* = 8) suggested lower prolidase activity in diabetic acute MI subjects compared with non-diabetic acute MI subjects (*p* = 0.02).

**Conclusion:**

Circulating prolidase is not significantly different between patients with acute MI and stable CAD or between patients with thrombotic and non-thrombotic MI. Further studies are required to determine if diabetes significantly affects prolidase activity and how this might relate to the risk of MI.

## Introduction

Acute myocardial infarction (MI) remains a leading cause of death worldwide (1). Plaque disruption with superimposed thrombosis is a hallmark of acute MI (2); however, the mechanisms leading to plaque disruption and those that determine the nature and the extent of ensuing thrombotic responses remain unclear (3). Particularly important to plaque stability is the strength of the fibrous cap, which depends on the balance between extracellular matrix (ECM) synthesis and degradation (4). Proteolytic enzymes, such as matrix metalloproteases (MMPs), have been identified in human coronary atherosclerotic plaques (5) and are known to mediate vascular remodeling by regulating degradation of ECM components (6). Furthermore, rupture-prone, thin-cap fibroatheromas express greater levels of MMPs than stable plaques having thicker fibrous composition (7–9) and are generally implicated in coronary atherosclerotic plaque destabilization (10–13).

Although different MMPs initiate the breakdown of collagen, the final step of collagen degradation and the initial step of collagen biosynthesis are mediated by prolidase (also termed peptidase D); thus, prolidase is considered the major rate-limiting step regulating collagen turnover (14). Because collagen is the major ECM constituent in atherosclerotic plaques, accounting for up to 60% of the total protein content (15), it is plausible that prolidase activity regulates plaque stability by modulating collagen turnover in atherosclerotic plaques. In support of this idea, prolidase has been shown to be significantly higher in patients with coronary artery disease (CAD) and prolidase activity has been positively associated with severity of CAD (16). Nevertheless, the impact of prolidase on plaque stability has not been assessed, and it remains unclear whether prolidase levels are predictive of acute MI.

In this study, we determined serum prolidase activity (SPA) at the time of acute MI compared with a quiescent state in the same individuals. As an additional control, changes in prolidase over the same time course were measured in patients with stable CAD undergoing cardiac catheterization. Because changes in prolidase activity were expected to be associated only with events precipitated by plaque disruption, we performed a subgroup analysis to evaluate prolidase activity in subjects presenting with thrombotic (type I) versus non-thrombotic (type II) MI. The MI type was determined using novel criteria specifically developed for this study and incorporated historical, biochemical, electrocardiographic, histological and core lab read coronary angiographic data. Additionally, because diabetics have higher rates of MI in general (17–19), we determined whether prolidase activity was associated with diabetes status in a small number of diabetic patients.

## Materials and Methods

### Enrollment Population

Following Institutional Review Board approval, participants were recruited from two hospitals in Louisville, Kentucky between March 2012 and August 2013 and were followed prospectively. Two types of subjects were sought for enrollment: those with suspected acute MI and those with suspected stable CAD. Subjects who received fibrinolytics were excluded. All participants provided written informed consent. This cohort was specifically designed to create three specific, non-overlapping phenotypes (acute thrombotic MI, acute non-thrombotic MI, stable CAD) to allow diagnostic and mechanistic investigation of acute thrombotic MI. This cohort and the criteria utilized to define the unique study groups (thrombotic MI and non-thrombotic MI) in this study have been utilized in prior work published by our laboratory in the investigation of analytes unrelated to this study [plasminogen (PLG) and oxidized phospholipids bound to PLG] (20). A description of this cohort, which has been previously published (20), is repeated here for completeness and convenience.

Enrollment criteria for both groups required that each patient be >18 years of age and scheduled for coronary angiography within 48 h. Those enrolled in the suspected acute MI group must have reported ischemic symptoms lasting >10 min, within 24 h of enrollment, and had to meet at least one of the following four criteria: (1) new or presumably new ST-segment depression >0.1 mV, (2) elevated troponins or CK-MB levels within 24 h of enrollment, (3) ≤1 mm ST-segment elevation in ≥2 contiguous electrocardiogram (ECG) leads, or (4) ≥1 mm ST-segment depression in V1 and V2 (posterior wall infarct). Subjects considered for enrollment in the suspected stable CAD group were required to have presented for angiography as an elective procedure. Patients in the suspected stable CAD group were excluded on the basis of any one of the following criteria: (1) hospitalization for acute coronary syndrome (ACS) or clinical instability within 4 weeks prior to planned enrollment, (2) coronary artery bypass grafting (CABG) surgery within 1 year prior to planned enrollment, (3) percutaneous coronary intervention (PCI) within 12 weeks prior to planned enrollment, (4) stroke within 12 weeks prior to planned enrollment, (5) presence of unstable angina or symptoms refractory to maximal medical therapy, (6) presence of significant co-morbidities likely to cause death within 2 years, or (7) significant active history of substance abuse within 5 years of enrollment. Subjects were asked to decline enrollment if they would not be able to return to the medical campus for a 3-month stable follow-up.

### History, Physical Exam, Clinical Laboratory, and ECG Data

All subjects were evaluated by study personnel, and each subject’s history, physical examination results, clinical laboratory data, and ECG data were collected prior to measurement of prolidase. The subject’s medical records were used to aid in the collection of pertinent medical history. A single study physician (Andrew P. DeFilippis) read all ECGs in accordance with *a priori* study guidelines. Standard laboratory data (troponin, creatinine, blood cell, and platelet counts) were obtained from the treating hospital clinical laboratory at standardized study time points: at the time of cardiac catheterization, prior to angiogram or any coronary intervention (T0), and 6 h post-angiogram (T6) (unless the subject was discharged from the hospital prior to this time point). Follow-up history, physical exam results, and laboratory data were collected at a single follow-up (Tf/u) visit 3 to 12 (median, 3.27) months after the procedure or hospitalization for acute MI, when the subject was in a stable condition.

### Biochemical Analyses

Enrollment sample collection *via* an arterial sheath took place at the time of cardiac catheterization, prior to coronary angiography, after a 5–10 ml waste draw. All available follow-up samples (T6 and >3 months) were collected from a peripheral vein, preferably a virgin vein, without a tourniquet (maximum pressure of <40 mmHg *via* blood pressure cuff), using a 21G needle, after >10 ml of clinical blood collection (waste draw), and into a tube containing ethylenediaminetetraacetic acid. Sample processing time was rigorously standardized. Serum obtained at the T0, T6, and Tf/u time points was frozen at −80°C until assay.

### Prolidase Assay

Prolidase activity in serum was determined using a colorimetric method based on the measurement of proline levels liberated enzymatically from an exogenous glycyl-l-proline (Gly-Pro) substrate. For this, we optimized the method described by Myara et al. (21) for use in a high-throughput plate reader. The following materials were used for measuring prolidase activity: Ninhydrin (Sigma 151173), Gly-Pro (Sigma G3002), manganese chloride (Sigma 244589), l-Proline (Sigma 81709), and reduced l-glutathione (GSH; Sigma G6013). Chinard’s reagent was prepared by dissolving 10 g ninhydrin in 240 ml of glacial acetic acid heated to 70°C, and then adding 66 ml of orthophosphoric acid, followed by 94 ml of distilled water. This stock reagent was stored at room temperature. A working solution of Chinard’s reagent was prepared by diluting the stock reagent with glacial acetic acid (1:1, v/v) immediately prior to assay. Briefly, 15 µl of serum from each patient and time point were added to 1.2 ml strip-tubes (8-strip cluster tubes), followed by addition of 85 µl of 50 mM Tris–HCl, pH 7.80, containing 20 mM MnCl_2_ and 1 mM GSH. In a preincubation step, which was required to activate the prolidase enzyme, this mixture was incubated for 1 h at 37°C. In standardization studies, we found this duration to be sufficient to activate the enzyme maximally. Next, we added 100 µl of 94 mM Gly-Pro in 50 mM Tris–HCl, pH 7.80, and incubated the solutions for 30 min at 37°C. Then, to stop the reaction, we added 0.8 ml of ice-cold, 0.45 M trichloracetic acid. The mixtures were then centrifuged for 15 min at 12,000 *g*, and 0.25 ml of supernatant was transferred into 1.7 ml Eppendorf tubes. To this, we added 1 ml of Chinard’s reagent working solution, and the samples were subsequently incubated for 12 min at 90°C. The solutions were then cooled on ice for 15 min, and 300 µl was transferred to wells of a 96-well plate, in triplicate. The absorbance was read at 515 nm and recorded using a BioTek Synergy 2 Multi-Mode plate reader. Each assay included a respective blank (300 µl of 0.25 ml TCA + 1 ml Chinard’s reagent working solution) as well as two separate quality control samples used to delineate process variability from assay to assay (sera from two donors). Also included was a standard (650 µM proline in 0.25 ml TCA + 1 ml Chinard’s working solution); standard curves constructed using 0–700 nM proline showed excellent linearity (*R*^2^ > 0.99).

Serum prolidase activity was calculated as in the study by Myara et al. (21), factoring in volumetric proportions of Chinard’s working solution and sample supernatant as well as appropriate dilution factors. The coefficient of variation (CV), calculated using quality control samples, was <2.5%. Human serum samples that showed evidence of hemolysis were omitted from the study.

### Serum Cardiac Troponin I Measurements

Troponin concentrations were measured by either of two independent CLIA-approved laboratories, the University of Louisville or KentuckyOne Jewish Hospital. The Ortho VITROS 5600 assay was used to measure cardiac troponin I in subjects receiving treatment at the University of Louisville Hospital. For this assay, the 99% cutoff level for a healthy population was 0.035 ng/ml with a (CV) <10%. This assay further defined 0.12 ng/ml as the most efficient (more specific) cutoff point for the diagnosis of acute MI. The Beckman Access assay was used to measure cardiac troponin I in subjects receiving treatment at KentuckyOne Jewish Hospital. For this assay, the 99% cutoff level for a healthy population was 0.04 ng/ml, but a CV < 10% was not achieved until 0.06 ng/ml. This assay defined 0.5 ng/ml as the most efficient (more specific) cutoff point for the diagnosis of acute MI.

### Coronary Angiographic Assessment

Angiograms were examined in a blinded fashion for all subjects by the Johns Hopkins Quantitative Angiographic Core Laboratory. The criteria for identifying and quantifying coronary thrombosis and atherosclerotic burden were jointly developed by the Core lab and the study team from existing published data (22–29).

### Histological Data

Coronary aspiration, with intent to retrieve the culprit coronary thrombosis, was left to the discretion of the subject’s treating interventional cardiologist. All samples from aspiration attempts were strained, immediately preserved in formalin, and sent to CVPath Institute, Inc., Gaithersburg, MD, USA, for blinded histological evaluation by a pathologist specialized in the analysis of coronary thrombosis (30, 31).

### Study Cohort

The following strict *a priori* criteria were used to define the study analysis groups.

**Figure 1:**
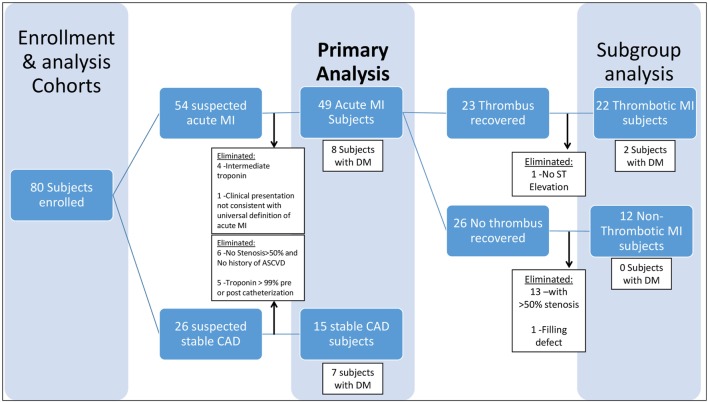
Enrollment and Analysis Cohorts.

*Acute MI* was defined as a clinical event in a subject presenting to the cardiac catheterization laboratory for a non-elective procedure, with a cardiac troponin I level greater than the most efficient diagnostic cutoff point as specified by the assay manufacturer (several-fold higher than the 99th percentile for a healthy population for both assays used in this study) and meeting Joint ESC/ACCF/AHA/WHF criteria (32) for an acute MI (Table 1). Patients with *stable CAD* were identified as those presenting for an elective cardiac catheterization with a past medical history of atherosclerosis, as evidenced by CABG, PCI, stroke/transient ischemic attack, carotid endarterectomy, peripheral artery bypass procedure, abdominal aortic aneurysm repair, or >50% stenosis in one or more coronary vessels on enrollment angiogram. Additional criteria included normal thrombolysis in myocardial infarction (TIMI) flow and myocardial perfusion grade (MPG) in all vessels as well as pre- and post-procedure cardiac troponin I <99% for a healthy population specific to the assay used (Table 1). Subjects who did not meet either stable CAD or acute MI criteria were eliminated from the study in order to limit obfuscation from misclassification and to produce an ideal cohort for discovering new biochemical/clinical characteristics related to acute MI (Figure 1).

**Table 1:** Study phenotype criteria.

Study phenotype	Criteria			
	Troponin (ng/ml)	Histology	Presentation	Blinded angiographic assessment
Stable CAD (*n* = 15)	Ortho VITROS 5600 Assay: “Peak” troponin level < 0.035 Beckman Access “Peak” troponin level < 0.04		Elective coronary angiogram History of CVD: CABG, PCI, CVA/TIA, CEA, PAD, or AAA procedure or angiographic criteria	Satisfies ALL criteria below: >50% stenosis in one or greater epicardial vessel (only required if no history of CVD) TIMI flow = 3 (all vessels) TIMI MPG = 3 (all vessels)

Acute MI (*n* = 49)	Ortho VITROS 5600 Assay: “Peak” troponin level > 0.12 Beckman Access “Peak” troponin level > 0.5		Non-elective coronary angiogram Clinical presentation consistent with WHF/ECC/ACC/AHA Universal definition of AMI

Thrombotic MI (*n* = 22)	Ortho VITROS 5600 Assay: “Peak” troponin level > 0.12 Beckman Access “Peak” troponin level >0.5 and >30% increase in troponin from T0 to T6	Histologically confirmed coronary thrombus 0–4 days old by blinded pathological assessment	Non-elective coronary angiogram Clinical presentation consistent with WHF/ECC/ACC/AHA Universal definition of AMI	Stenosis in the vessel in which thrombus was recovered of 50–100% and ST elevation in territory supplied by the vessel in which thrombus was recovered

Non-thrombotic MI (*n* = 12)	Ortho VITROS 5600 Assay: “Peak” troponin level >0.12 Beckman Access “Peak” troponin level >0.5	No histologically confirmed thrombus recovered	Clinical presentation consistent with WHF/ECC/ACC/AHA Universal definition of AMI	Satisfies ALL 5 criteria below, in all vessels: <50% stenosis No filling defect Simple Ambrose lesion morphology TIMI flow = 3 TIMI MPG = 3

*CAD, coronary artery disease; MI, myocardial infarction; TIMI, thrombolysis in myocardial infarction; MPG, myocardial perfusion grade; CABG, coronary artery bypass grafting; PCI, percutaneous coronary intervention; CVA, cerebral vascular accident; TIA, transient ischemic attack; CEA, carotid endarterectomy; PAD, peripheral artery disease; AAA, abdominal aortic aneurysm; CVD, cardiovascular disease*.

Acute MI was further stratified into thrombotic (Type 1) and non-thrombotic (Type 2) MI. Because no national guidelines exist for differentiating thrombotic (Type 1) and non-thrombotic (Type 2) MI, we developed novel conservative (stringent) criteria (Table 1) to eliminate borderline cases from our analysis and limit confounding from misclassification, with the goal of identifying biological characteristics related to acute thrombotic MI (Figure 1). Our criteria expand upon those previously proposed by our group (33) and now include a combination of prospectively collected historical, physical, electrocardiographic, histological, biochemical, and angiographic (blinded core lab assessment) data that we believe are more robust than any other published criteria for distinguishing Type 1 and Type 2 MI (34–40). The definition of *thrombotic MI* included the criteria for acute MI as well as (1) the presence of a histologically confirmed (by blinded pathological assessment) coronary thrombus 0–4 days old, (2) a 50–100% stenosis in the vessel in which the thrombus was recovered, (3) ST elevation in the territory supplied by the vessel in which the thrombus was recovered, and (4) an elevated troponin and >30% increase in troponin between T0 and T6 hours (Table 1). *Non-thrombotic MI* was defined as meeting the criteria for acute MI, but with (1) no recovery of a histologically confirmed thrombus and (2) satisfaction of all of the following five criteria in all vessels: <50% stenosis, no filling defect, simple Ambrose lesion morphology, TIMI flow = 3, and TIMI myocardial perfusion grade (TIMI MPG) = 3 (Table 1). *Diabetes mellitus* was defined as (1) fasting glucose of more than 126 mg/dl and/or (2) HgA1C > 6.5 and/or (3) patients on hypoglycemic medications (insulin, oral anti-hyperglycemic medications).

### Statistical Analysis

Frequencies, percentages, and Fisher’s exact test *p*-values are reported for comparing the distribution of categorical characteristics across study groups. Mean, SD, and appropriate statistical test *p*-values (Student’s *t*-test, Welch’s *t*-test, or Wilcoxon rank-sum test) are reported for continuous variable comparisons. Prolidase activity was log-transformed to ensure that the outcome measure was normally distributed for further analyses. To identify covariates that were associated with prolidase activity (potential confounding factors), the signed Pearson correlation coefficient was computed for relevant covariates at the quiescent phase (Tf/u). The primary analysis consisted of comparing prolidase activity across study time points between acute MI subjects and stable CAD subjects. A secondary analysis was conducted to compare prolidase activity between MI subgroups (thrombotic MI versus non-thrombotic MI). A repeated measures analysis of variance (RM-ANOVA) was conducted for both the primary and secondary analyses. For the RM-ANOVA, linear models were constructed with fixed effects for study group, time point, group × time point interaction, and a random effect for study subject. A compound symmetry covariance structure was assumed for the study subject random effect. If evidence of a group × time point interaction was not found, this interaction was removed. Confounding factors were considered for incorporation in the RM-ANOVA. The inclusion of these fixed effects allowed for testing: (1) whether mean prolidase levels differed by study group, (2) whether prolidase levels differed across time points, and (3) whether time course differed by study group. The *p*-values from Type III SS *F*-tests are reported for determining statistical significance. For the primary analysis comparing the acute MI and stable CAD study groups, T0, T6, and Tf/u measurements were used. For the subgroup analysis comparing the thrombotic (Type 1) MI and non-thrombotic (Type 2) MI subgroups, T0, T6, and Tf/u measurements were used. The random effect for study subject assumed a compound symmetry covariance structure. Statistical power for both the primary and acute MI subgroup analyses was retrospectively calculated using the observed analyte means, variances, and group sizes (41). To identify potential effect modifiers in the repeated measures analysis, the relationships between the analytes and cohort characteristics were examined. Signed *r*^2^, where *r* is the Pearson correlation coefficient, are presented to show the magnitude, direction, and significance of associations between a characteristic and an analyte. Further statistical modeling was conducted to evaluate characteristics identified as effect modifiers. Statistical analyses were conducted in R version 3.2 (R Core Team, 2015). The manuscript is in compliance with the STROBE (& MOOSE) guidelines for observational studies.

## Results

A total of 49 patients met the criteria for an acute MI, of which a subset had clearly defined thrombotic MI (*n* = 22) or non-thrombotic MI (*n* = 12), and 15 patients met the criteria for stable CAD (Table 1). Of the 49 patients in the acute MI group, 16.3% (*n* = 8) were diabetic, and out of 15 patients in the stable CAD group, 46.7% (*n* = 7) had DM. Excluding constrained differences resulting from the enrollment criteria, the prevalence of smoking and heart rate on presentation was higher among acute MI subjects, whereas body mass index (BMI) and prevalence of hyperlipidemia and diabetes were higher among the subjects with stable CAD (Table 2). Excluding constrained differences resulting from the criteria employed to adjudicate thrombotic MI versus non-thrombotic MI, the time from presentation to angiogram, the peak troponin, and the baseline glucose values differed between individuals in the thrombotic MI group compared with those in the non-thrombotic MI group (Table 3).

**Table 2:** Cohort characteristics.

Variable	Acute MI group (*N* = 49)	Stable CAD group (*N* = 15)	*p*-Value
Age (mean ± SD) in years	58.6 ± 14.4	61.3 ± 8.9	0.49
Males (%)	65.3	53.3	0.54
Caucasian race (%)	77.6	93.3	0.09
Current smoker (%)	53.1	20.0	0.04
Former smoker (%)	26.5	60.0	0.04
Currently consumes alcohol (%)	32.7	46.7	0.37
History of dyslipidemia (%)	46.9	86.7	0.01
History of diabetes mellitus (%)	16.3	46.7	0.03
History of hypertension (%)	61.2	93.3	0.06
History of atherosclerosis (%) (MI, CAD, PCI, CABG)	38.8	100.0	<0.0001
History of congestive heart failure (%)	8.2	6.7	1.00
History of chronic renal failure (%)	8.2	0.0	0.28
History of stroke (%)	10.2	0.0	0.33
HR at time of presentation (mean ± SD)	84.9 ± 25.0	65.9 ± 9.6	<0.0001
MAP at time of presentation (mean ± SD)	102.8 ± 24.7	91.4 ± 14.3	0.09
BMI at time of presentation (mean ± SD)	27.5 ± 7.24	33 ± 7.08	0.02
Time (hours) elapsed presentation to T0 (median ± IQR, range)	3.5 ± 16.0, 36	2.0 ± 1.0, 3	0.21^b^
Glucose at baseline (mean ± SD, range)	142.1 ± 56.5, 285	131.6 ± 30.6, 107	0.35^a^
Creatinine at baseline (mean ± SD, range)	1.13 ± 0.79, 4.34	0.92 ± 0.17, 0.66	0.10^a^
ST elevation on EKG at baseline	63.3	0.0	<0.0001
One vessel with ≥50% coronary stenosis on enrollment angiogram	79.6	66.7	0.31
Aspirin use at time of enrollment (%)	89.8	86.7	0.66
P2Y12 inhibitors use at enrollment (%)	53.1	50.0	0.77

*^a^Welch’s t-test*.

*^b^Wilcoxon rank-sum test*.

*MI, myocardial infarction; CAD, coronary artery disease; PCI, percutaneous coronary intervention; CABG, coronary artery bypass grafting; BMI, body mass index*.

**Table 3:** Baseline subject characteristics for acute MI subgroup analysis.

Variable	Type 1 MI (*N* = 22)	Type 2 MI (*N* = 12)	*p-*Value
Age (mean ± SD) years	58.7 ± 15.1	56.3 ± 16.6	0.67
Males (%)	72.7	41.7	0.14
Caucasian race (%)	86.4	66.7	0.21
Current smoker (%)	45.5	50.0	1.00
Currently consumes alcohol (%)	36.4	33.3	0.78
History of dyslipidemia (%)	54.5	33.3	0.30
History of diabetes mellitus (%)	9.1	0.0	0.53
History of hypertension (%)	45.5	75.0	0.19
History of atherosclerosis (%) (MI, CAD, PCI, CABG)	40.9	33.3	0.73
History of congestive heart failure (%)	0.0	8.3	0.35
History of chronic renal failure (%)	4.5	8.3	0.43
History of stroke (%)	4.5	25.0	0.12
HR at time of presentation (mean ± SD)	87.4 ± 27.2	88.3 ± 27.5	0.93
MAP at time of presentation (mean ± SD)	104.1 ± 19.8	95.4 ± 26.9	0.29
BMI at time of presentation (mean ± SD)	27.5 ± 7.4	27.8 ± 6.7	0.90
Time (hours) elapsed presentation to angiogram (median ± IQR, range)	1.0 ± 1.0, 13.0	18.0 ± 9.5, 30.0	<0.0001^b^
Baseline troponin (mean ± SD, range) mg/dL	7.8 ± 14.9, 55.3	2.4 ± 2.9, 9.8	0.76^b^
Peak troponin (mean ± SD, range)	51.6 ± 41.2, 145.7	11.6 ± 22.0, 68.2	0.0002^b^
Glucose at baseline (mean ± SD, range)	166.5 ± 58.9, 246	118.0 ± 47.7, 182	0.01^a^
Creatinine at baseline (mean ± SD, range)	0.98 ± 0.43, 2.38	1.21 ± 1.07, 3.80	0.50^a^
ST elevation on EKG at baseline	100.0	25.0	<0.0001
One vessel with ≥50% coronary stenosis on enrollment angiogram	100.0	0.0	<0.0001
Aspirin use at time of enrollment (%)	86.4	91.7	1.00
P2Y12 inhibitors use at enrollment (%)	45.5	50.0	1.00

*^a^Welch’s t-test*.

*^b^Wilcoxon rank-sum test*.

*MI, myocardial infarction; CAD, coronary artery disease; PCI, percutaneous coronary intervention; CABG, coronary artery bypass grafting; HR, heart rate; SBP, systolic blood pressure; DBP, diastolic blood pressure; MAP, mean arterial pressure; BMI, body mass index; ACE-I, angiotensin converting enzyme inhibitor*.

### Primary Analysis

**Figure 2:**
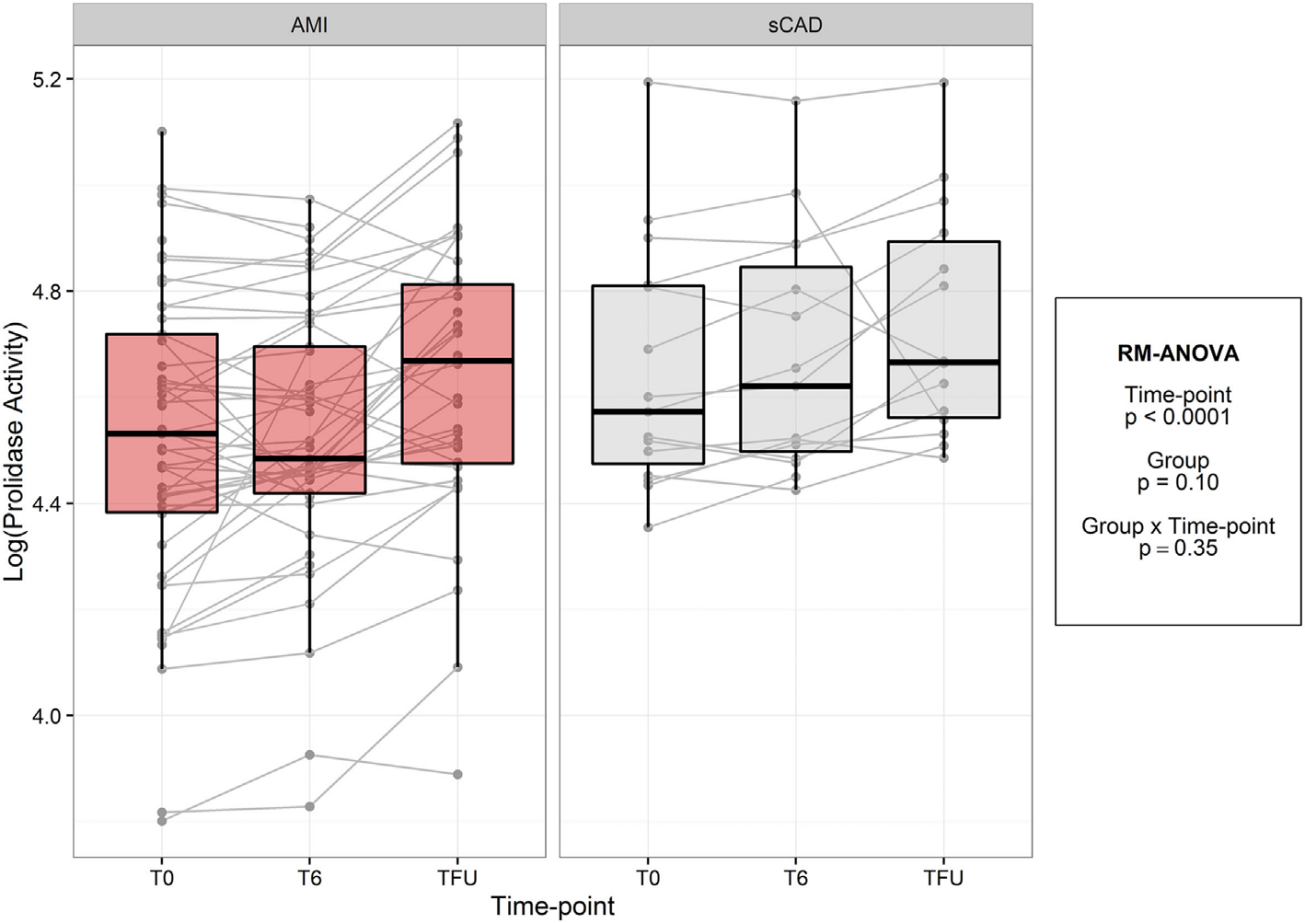
Log-transformed prolidase activity across study time points by group. Units are in log mmol/l/min.

**Figure 3:**
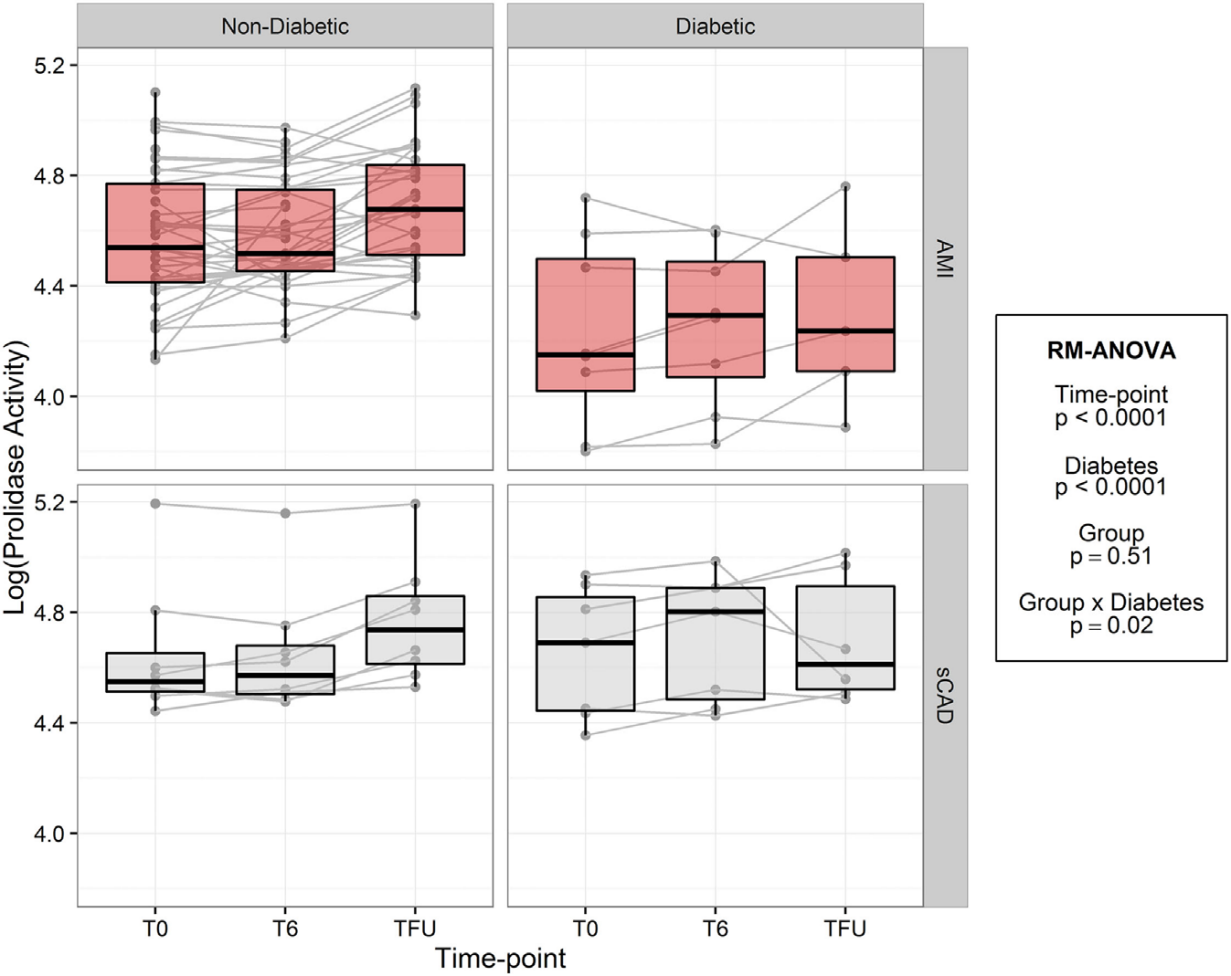
Log-transformed prolidase activity across study time points by group and diabetes. Units are in log mmol/l/min.

In both acute MI and stable CAD subjects, mean prolidase activity was lower at acute phase time points (T0, T6) versus the quiescent phase (Tf/u) (RM-ANOVA time point *p* < 0.0001) (Figure 2). There was not a statistically significant difference in prolidase activity at any time point between acute MI and stable CAD subjects (*p* = 0.35). Potential confounding by risk factors including smoking, heart rate on presentation, BMI, history of hyperlipidemia, time from presentation to angiogram, the peak troponin, and baseline glucose was evaluated by adding each of these terms individually to the multivariable model. Each risk factor was non-significant (*p* > 0.05) and did not improve model fit (*F*-test, *p* > 0.05). These finding suggest that practices related to cardiac catheterization may lower circulating prolidase activity acutely. Nevertheless, prolidase activity at the quiescent state was predictive of prolidase activity in the acute state (T0) (*R*^2^ = 0.71, *p* < 0.0001) (Table 4). Prolidase activity was also significantly correlated with DM at the time point representing the quiescent state (*R*^2^ = 0.10, *p* = 0.03); however, it was not correlated with serum glucose at follow-up (Tf/u) (Table 4). In a stratified analysis, mean prolidase activity was lower among diabetic acute MI subjects compared with non-diabetic acute MI subjects [*p* < 0.05 as evident from non-overlapping 95% CI of the mean (Table 5) and diabetes effect *p* < 0.0001; MI × diabetes *p* = 0.02 (Figure 3)]. Similarly, mean prolidase activity was lower among diabetic acute MI subjects compared with both diabetic and non-diabetic stable CAD subjects at both the acute and quiescent time points [*p* < 0.05 as evident from non-overlapping 95% CI of the mean (Table 5) and diabetes effect *p* < 0.0001; MI × diabetes *p* = 0.02 (Figure 3)]. No significant differences in prolidase activity were observed between the acute (T0, T6) and quiescent (Tf/u) states in any of the strata (non-diabetic acute MI, non-diabetic stable CAD, diabetic acute MI, diabetic stable CAD) (Table 5). However, when all groups were analyzed together, prolidase activity was lower at the acute time points (T0, T6) compared with the quiescent state (Tf/u) (RM-ANOVA time point *p* < 0.0001), and there was marginal evidence that this relationship was modified by the presence or absence of acute MI (*p* = 0.10) (Figure 2). Collectively, these data suggest that prolidase activity is generally diminished at the time of cardiac catheterization and that prolidase activity may be lower in diabetic MI patients.

**Table 4:** Association of covariates and prolidase activity at quiescent state.

	Prolidase (F/U)^a^	
Variable	Signed *R*^2^	*p*-Value
Prolidase (enrollment)^a^	0.71	**<***0.0001*****
Glucose (F/U)^a^	−0.08	**0.06**
Creatinine (F/U)^a^	−0.08	***0.05***
Gender (female versus male)	0.03	0.19
Diabetes	0.10	***0.03***
Lipidemia	0.01	0.46
Smoking	0.00	0.74
Alcohol	0.02	0.29
PCI	0.01	0.45
Race (Caucasian versus non-Caucasian)	0.01	0.58
SBP	0.05	0.09
DBP	0.03	0.15
MAP	0.04	0.11
HR	−0.06	**0.06**
RR	0.00	0.65
BMI	0.00	0.90
Age	0.00	0.78

*^a^Log-transformed*.

*PCI, percutaneous coronary intervention; BMI, body mass index*.

*Variables in bold italic which were statistically significant*.

*Variable in bold which has some association with prolidase but did not achieve statistical significance*.

**Table 5:** Mean prolidase activity and 95% confidence intervals for mean prolidase activity by group and diabetes.

	Non-diabetic		Diabetic	
Time point	Acute MI (*n* = 41)	Stable CAD (*n* = 8)	Acute MI (*n* = 8)	Stable CAD (*n* = 7)
T0	97.8 (90.9, 105.2)	103.6 (88.2, 121.5)	69.0 (58.7, 81.0)	102.8 (86.6, 121.9)
T6	100.6 (93.4, 108.3)	106.5 (90.7, 125.0)	70.9 (60.4, 83.3)	105.7 (89.1, 125.4)
TFU	109.5 (101.6, 118.0)	115.9 (98.8, 136.1)	77.2 (65.7, 90.8)	115.1 (96.9, 136.6)

*Units are mmol/l/min*.

*MI, myocardial infarction; CAD, coronary artery disease*.

### Exploratory Analysis

**Figure 4:**
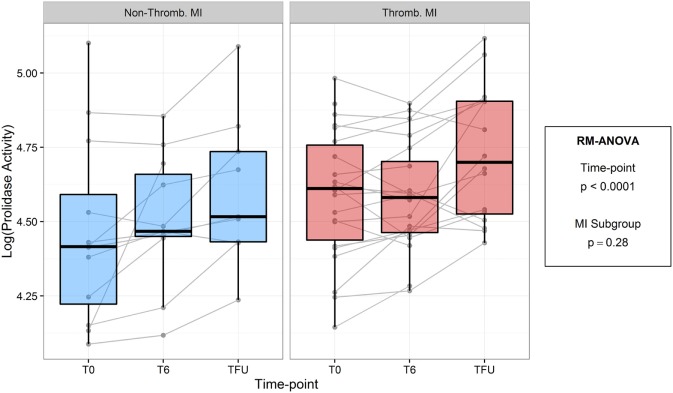
Log-transformed prolidase activity across study time points by myocardial infarction (MI) subgroup. Units are in log mmol/l/min.

To test the hypothesis that prolidase activity is acutely associated with plaque stability and acute plaque disruption, we compared prolidase activity in thrombotic and non-thrombotic acute MI subjects (Figure 4; Table 6). We did not observe evidence of a difference in prolidase activity between thrombotic MI versus non-thrombotic MI subjects at any acute (T0, T6) or quiescent time point (*p* = 0.28). Potential confounding variables, i.e., baseline glucose, times from presentation to angiogram and peak troponin, were evaluated by adding each of these terms individually to the multivariable model. Each risk factor was non-significant (*p* > 0.05) and did not improve model fit (*F*-test, *p* > 0.05). The sample size was not adequate to stratify thrombotic and non-thrombotic MI subgroups by diabetes status.

**Table 6:** Model estimated mean prolidase activity and 95% confidence intervals for mean prolidase activity by MI subgroup.

Time point	Non-thrombotic MI (*n* = 12)	Thrombotic MI (*n* = 22)
T0	88.8 (77.5, 101.9)	97.2 (87.7, 107.7)
T6	92.5 (80.6, 106.2)	101.2 (91.2, 112.2)
TFU	102.9 (89.5, 118.3)	112.6 (101.3, 125.1)

*Units are mmol/l/min*.

*MI, myocardial infarction*.

## Discussion

To our knowledge, this is the first study to measure the activity of prolidase in patients with acute MI. Stimulated by the fact that plaque stability is in part regulated by collagen and fibrous cap strength (7–9), we addressed whether prolidase—a major contributor to collagen turnover—differs in patients with acute MI. We found that prolidase activity was lower at the time of presentation for acute MI as compared with a quiescent (>3 months) follow-up. However, low prolidase activity was also observed in stable CAD subjects undergoing elective cardiac catheterization and did not differ between acute thrombotic versus acute non-thrombotic MI subjects, suggesting that prolidase activity is impacted by practices related to cardiac catheterization rather than acute MI itself. Although prolidase activity was highly correlated with diabetes and found to be significantly lower compared with acute MI subjects without diabetes or with subjects with stable CAD (with or without diabetes), the small number of diabetic patients precludes us from deriving strong conclusions. Overall, we conclude that circulating prolidase is not significantly different between patients with acute MI and stable CAD or between patients with thrombotic and non-thrombotic MI. Further studies are needed to determine if diabetes significantly affects prolidase activity and how changes in prolidase activity may relate to the risk of MI.

Although we found no significant differences in SPA between acute MI and stable CAD subjects undergoing cardiac catheterization, the mean SPA was lower at the time of presentation/procedure as compared to quiescent follow-up. This finding suggests that the lower SPA levels at the time of acute MI may be more reflective of the acute stress or specifics of the cardiac catheterization procedure rather than plaque disruption. Although it is unclear why prolidase activity was lower at the early time points, peri-procedure use of medications, particularly use of antiplatelet agents and anticoagulants, may affect SPA. Indeed, Surazynski et al. showed that acetylsalicylic acid decreases collagen biosynthesis by decreasing prolidase activity (42). Similarly, Demirtas et al. found SPA to be significantly lower in subjects who received anticoagulants and antiplatelet agents compared with controls (43). Antiplatelet, anticoagulant, or other medications affecting the collagen cycle, like enalaparil, have also been shown to affect prolidase activity (44). Because almost all patients received aspirin, and patients that had PCI received dual antiplatelet therapy along with heparin, which has anticoagulant and antiplatelet properties, it is possible that medications provided at the time of cardiac catheterization contributed to the generally lower levels of prolidase activity at the early time points (T0 and T6).

One aim of our study was to determine whether prolidase activity differs among subjects with acute thrombotic MI versus acute non-thrombotic MI. Our findings indicate that prolidase activity is not different between patients with thrombotic and non-thrombotic MI; nor was it different between acute MI subjects and subjects with stable CAD. However, the fact that prolidase activity is correlated with other pathological conditions such as diabetes provided us with rationale to perform further, stratified analyses. Prolidase activity has been measured in tissues such as plasma, erythrocytes, leukocytes, and dermal fibroblasts (45, 46) in conditions including chronic liver disease (21), osteoporosis (47), osteoarthritis (48), DM (47), and uremia (49). Among cardiovascular conditions, SPA was shown to be independently associated with severity of left ventricular diastolic dysfunction (50), hypertension (51), coronary artery aneurysms (52), aortic dilation (53), slow coronary flow (54), and presence of CAD (16). Sezen et al. showed that prolidase activity was significantly lower in patients with dilated cardiomyopathy relative to healthy volunteers, and lower in subjects with ischemic cardiomyopathy compared with those diagnosed with idiopathic cardiomyopathy (55).

Our finding that diabetic subjects with acute MI have low levels of SPA could be important because diabetics have a disproportionately higher rate of acute MI: 33% of acute MI occurs in diabetics, and diabetes is associated with a >2-fold increase in recurrent major adverse cardiac events after index MI compared with non-diabetic counterparts (17–19). Furthermore, diabetics are known to have generalized defects in connective tissue metabolism. For example, it has been shown that insulin and/or insulin-like growth factors and their coordinate signaling, which is defective in insulin-resistant states such as diabetes, impact collagen metabolism (56–58). While prolidase levels appear to be lower among diabetic patients in general (47, 59), prolidase activity was reported to be higher among diabetics with neuropathy (60), foot ulcers (61), and microalbuminuria (62). Our study might suggest the existence of a subgroup of diabetic patients that are susceptible to MI because of defective prolidase levels or activity. This hypothesis would be consistent with studies indicating an increased risk of MI in diabetics versus non-diabetics (63–67). Whether low prolidase activity in diabetics contributes to atherothrombosis, plaque rupture, or impaired plaque healing after rupture warrants further investigation.

### Strengths

Addressing our hypothesis required a unique cohort of acute MI subjects that included a subset of patients with acute thrombotic and non-thrombotic MI as well as a rigorous and high-throughput method for measuring prolidase. We introduced optimized conditions for measuring prolidase activity in serum and included appropriate controls to assess day-to-day variability. Our findings suggest that prolidase activity is likely affected by conditions of cardiac catheterization and that it is not different in patients with acute MI compared with stable coronary CAD; however, our data from a small number of diabetic MI patients warrant further studies to determine whether low prolidase activity is a cause of MI and the extent to which is contributes to atherothrombosis.

### Limitations

Although this is the largest study of prolidase activity in subjects with acute MI to date, the sample size remains limited, particularly in the exploratory subgroup analyses of thrombotic (type 1) and non-thrombotic (type 2) MI subjects. Even though prolidase activity was found to be significantly diminished in diabetic patients with MI, the small sample size of diabetic patients precludes us from making strong conclusions regarding changes in prolidase as they relate to diabetes and MI. Thus, future studies with a higher sample size will be required to determine with confidence if circulating prolidase activity is lower in diabetic patients and if it might contribute to MI in patients with metabolic disease. Also, our study does not have long-term follow-up data on cardiovascular events and is therefore unable to evaluate prolidase as a predictor of events in stable CAD and post-MI patients. We measured prolidase activity in serum, which is a repository for metabolites, secreted proteins, and RNA/DNA from all tissues, but these measurements cannot indicate defects in prolidase activity or secretion from specific tissues. Therefore, it remains unclear whether significant differences in SPA indicate defective collagen remodeling at sites of unstable atherosclerotic lesions.

## Conclusion

In summary, we report that there is no significant difference in prolidase activity in patients with acute MI or stable CAD; however, we find that prolidase activity is lower at the time of cardiac catheterization compared to quiescent state follow-up. Our preliminary data from diabetic patients suggest prolidase activity is lower in diabetics with acute MI compared with non-diabetics with acute MI and stable CAD subjects (diabetic and non-diabetic) at both the acute and quiescent time points. These findings warrant further study to determine whether diabetes indeed diminishes circulating prolidase activity and whether insufficient prolidase activity contributes to the greater risk of MI in patients with diabetes.

## Ethics Statement

The study complies with the Declaration of Helsinki, the locally appointed ethics committee at University of Louisville has approved the research protocol, and informed consent has been obtained from all study subjects. No authors have any significant relationship with industry related to this work. All the authors have read and approved submission of the manuscript and the manuscript has not been published and is not being considered for publication elsewhere in whole or part in any language.

## Author Contributions

AS and YZ are the shared first authors and responsible for literature search and manuscript writing. PT helped with data analysis. YS and AA helped with data collection. BH and AD are the shared last authors and were responsible for cohort building, overseeing the data collection, analysis, and manuscript editing.

## Conflict of Interest Statement

Coronary aspiration material was evaluated at CVPath Institute, Inc., Gaithersburg, MD, USA. Coronary angiography was evaluated at the Johns Hopkins Quantitative Angiographic Core Laboratory, Baltimore, MD, USA. No author has any relationships with industry pertinent to this work.

## Abbreviations

AAA, abdominal aortic aneurysm; ACS, acute coronary syndrome; ApoB, apolipoprotein B; BP, blood pressure; CABG, coronary artery bypass grafting; CAD, coronary artery disease; CEA, carotid endarterectomy; CVD, cardiovascular disease; EDTA, ethylenediaminetetraacetic acid; ELISA, enzyme-linked immunosorbent assay; ECG, electrocardiogram; LBBB, left bundle branch block; MI, myocardial infarction; MPG, myocardial perfusion grade; PAI-1, plasminogen activator inhibitor-1; PCI, percutaneous coronary intervention; PLG, plasminogen; STEMI, ST-segment elevation myocardial infarction; SPA, serum prolidase activity; TIA, transient ischemic attack; TIMl, thrombolysis in myocardial infarction; tPA, tissue plasminogen activator.

## References

[1] Roger VL Go AS Lloyd-Jones DM Benjamin EJ Berry JD Borden WB Executive summary: heart disease and stroke statistics – 2012 update: a report from the American Heart Association. Circulation (2012) 125(1):188–97. 10.1161/CIR.0b013e3182456d46 22215894

[2] Libby P Molecular bases of the acute coronary syndromes. Circulation (1995) 91(11):2844–50. 10.1161/01.CIR.91.11.2844 7758192

[3] Arbab-Zadeh A Fuster V The myth of the “vulnerable plaque”: transitioning from a focus on individual lesions to atherosclerotic disease burden for coronary artery disease risk assessment. J Am Coll Cardiol (2015) 65(8):846–55. 10.1016/j.jacc.2014.11.041 25601032 PMC4344871

[4] Halvorsen B Otterdal K Dahl TB Skjelland M Gullestad L Oie E Atherosclerotic plaque stability – what determines the fate of a plaque? Prog Cardiovasc Dis (2008) 51(3):183–94. 10.1016/j.pcad.2008.09.001 19026853

[5] Galis ZS Sukhova GK Lark MW Libby P. Increased expression of matrix metalloproteinases and matrix degrading activity in vulnerable regions of human atherosclerotic plaques. J Clin Invest (1994) 94(6):2493–503. 10.1172/JCI117619 7989608 PMC330083

[6] Dollery CM McEwan JR Henney AM Matrix metalloproteinases and cardiovascular disease. Circ Res (1995) 77(5):863–8. 10.1161/01.RES.77.5.863 7554139

[7] Sukhova GK Schonbeck U Rabkin E Schoen FJ Poole AR Billinghurst RC Evidence for increased collagenolysis by interstitial collagenases-1 and -3 in vulnerable human atheromatous plaques. Circulation (1999) 99(19):2503–9. 10.1161/01.CIR.99.19.2503 10330380

[8] Libby P. Collagenases and cracks in the plaque. J Clin Invest (2013) 123(8):3201–3. 10.1172/JCI67526 23908120 PMC3726161

[9] Lenglet S Mach F Montecucco F. Role of matrix metalloproteinase-8 in atherosclerosis. Mediators Inflamm (2013) 2013:659282. 10.1155/2013/659282 23365489 PMC3556866

[10] Newby AC. Metalloproteinase expression in monocytes and macrophages and its relationship to atherosclerotic plaque instability. Arterioscler Thromb Vasc Biol (2008) 28(12):2108–14. 10.1161/ATVBAHA.108.173898 18772495

[11] Lehrke M Greif M Broedl UC Lebherz C Laubender RP Becker A MMP-1 serum levels predict coronary atherosclerosis in humans. Cardiovasc Diabetol (2009) 8:50. 10.1186/1475-2840-8-50 19751510 PMC2754422

[12] Cavusoglu E Marmur JD Hegde S Yanamadala S Batuman OA Chopra V Relation of baseline plasma MMP-1 levels to long-term all-cause mortality in patients with known or suspected coronary artery disease referred for coronary angiography. Atherosclerosis (2015) 239(1):268–75. 10.1016/j.atherosclerosis.2015.01.003 25635325 PMC4331241

[13] Hansson GK Libby P Tabas I. Inflammation and plaque vulnerability. J Intern Med (2015) 278(5):483–93. 10.1111/joim.12406 26260307 PMC5082111

[14] Surazynski A Miltyk W Palka J Phang JM Prolidase-dependent regulation of collagen biosynthesis. Amino Acids (2008) 35(4):731–8. 10.1007/s00726-008-0051-8 18320291

[15] Lijnen HR. Metalloproteinases in development and progression of vascular disease. Pathophysiol Haemost Thromb (2003) 33(5–6):275–81. 10.1159/000083814 15692229

[16] Yildiz A Demirbag R Yilmaz R Gur M Altiparmak IH Akyol S The association of serum prolidase activity with the presence and severity of coronary artery disease. Coron Artery Dis (2008) 19(5):319–25. 10.1097/MCA.0b013e32830042ba 18607169

[17] Nesto R C-reactive protein, its role in inflammation, Type 2 diabetes and cardiovascular disease, and the effects of insulin-sensitizing treatment with thiazolidinediones. Diabet Med (2004) 21(8):810–7. 10.1111/j.1464-5491.2004.01296.x 15270782

[18] Cavender MA Steg PG Smith SC Jr Eagle K Ohman EM Goto S Impact of diabetes mellitus on hospitalization for heart failure, cardiovascular events, and death: outcomes at 4 years from the reduction of atherothrombosis for Continued Health (REACH) Registry. Circulation (2015) 132(10):923–31. 10.1161/CIRCULATIONAHA.114.014796 26152709

[19] De Luca G Sauro R Capasso M Lanzillo T Manganelli F Carbone G Impact of diabetes on the benefits from everolimus-eluting stent as compared to first-generation drug-eluting stent in patients with ST elevation myocardial infarction. Diab Vasc Dis Res (2015) 12(5):306–14. 10.1177/1479164115592252 26150193

[20] DeFilippis AP Chernyavskiy I Amraotkar AR Trainor PJ Kothari S Ismail I Circulating levels of plasminogen and oxidized phospholipids bound to plasminogen distinguish between atherothrombotic and non-atherothrombotic myocardial infarction. J Thromb Thrombolysis (2016) 42(1):61–76. 10.1007/s11239-015-1292-5 26510751 PMC5403145

[21] Myara I Myara A Mangeot M Fabre M Charpentier C Lemonnier A. Plasma prolidase activity: a possible index of collagen catabolism in chronic liver disease. Clin Chem (1984) 30(2):211–5. 6692525

[22] Ambrose JA Almeida OD Sharma SK Dangas G Ratner DE. Angiographic evolution of intracoronary thrombus and dissection following percutaneous transluminal coronary angioplasty (the Thrombolysis and Angioplasty in Unstable Angina [TAUSA] trial). Am J Cardiol (1997) 79(5):559–63. 10.1016/S0002-9149(96)00815-6 9068508

[23] Ambrose JA Almeida OD Sharma SK Torre SR Marmur JD Israel DH Adjunctive thrombolytic therapy during angioplasty for ischemic rest angina. Results of the TAUSA Trial. TAUSA Investigators. Thrombolysis and angioplasty in unstable angina trial. Circulation (1994) 90(1):69–77. 10.1161/01.CIR.90.1.69 8026054

[24] Ambrose JA Israel DH. Angiography in unstable angina. Am J Cardiol (1991) 68(7):78b–84b. 10.1016/0002-9149(91)90388-2 1892071

[25] Capone G Wolf NM Meyer B Meister SG. Frequency of intracoronary filling defects by angiography in angina pectoris at rest. Am J Cardiol (1985) 56(7):403–6. 10.1016/0002-9149(85)90875-6 4036819

[26] Gibson CM Cannon CP Murphy SA Marble SJ Barron HV Braunwald E. Relationship of the TIMI myocardial perfusion grades, flow grades, frame count, and percutaneous coronary intervention to long-term outcomes after thrombolytic administration in acute myocardial infarction. Circulation (2002) 105(16):1909–13. 10.1161/01.CIR.0000014683.52177.B5 11997276

[27] Gibson CM Cannon CP Murphy SA Ryan KA Mesley R Marble SJ Relationship of TIMI myocardial perfusion grade to mortality after administration of thrombolytic drugs. Circulation (2000) 101(2):125–30. 10.1161/01.CIR.101.2.125 10637197

[28] Goldstein JA Demetriou D Grines CL Pica M Shoukfeh M O’Neill WW. Multiple complex coronary plaques in patients with acute myocardial infarction. N Engl J Med (2000) 343(13):915–22. 10.1056/NEJM200009283431303 11006367

[29] Zack PM Ischinger T Aker UT Dincer B Kennedy HL. The occurrence of angiographically detected intracoronary thrombus in patients with unstable angina pectoris. Am Heart J (1984) 108(6):1408–12. 10.1016/0002-8703(84)90684-7 6507234

[30] Kramer MC Rittersma SZ de Winter RJ Ladich ER Fowler DR Liang YH Relationship of thrombus healing to underlying plaque morphology in sudden coronary death. J Am Coll Cardiol (2010) 55(2):122–32. 10.1016/j.jacc.2009.09.007 19818571

[31] Kramer MC van der Wal AC Koch KT Ploegmakers JP van der Schaaf RJ Henriques JP Presence of older thrombus is an independent predictor of long-term mortality in patients with ST-elevation myocardial infarction treated with thrombus aspiration during primary percutaneous coronary intervention. Circulation (2008) 118(18):1810–6. 10.1161/CIRCULATIONAHA.108.780734 18852369

[32] Thygesen K Alpert JS Jaffe AS Simoons ML Chaitman BR White HD Third universal definition of myocardial infarction. J Am Coll Cardiol (2012) 60(16):1581–98. 10.1016/j.jacc.2012.08.001 22958960

[33] DeFilippis AP Oloyede OS Andrikopoulou E Saenger AK Palachuvattil JM Fasoro YA Thromboxane A(2) generation, in the absence of platelet COX-1 activity, in patients with and without atherothrombotic myocardial infarction. Circ J (2013) 77(11):2786–92. 10.1253/circj.CJ-12-1421 23985963

[34] Ambrose JA Loures-Vale A Javed U Buhari CF Aftab W Angiographic correlates in type 1 and 2 MI by the universal definition. JACC Cardiovasc Imaging (2012) 5(4):463–4. 10.1016/j.jcmg.2011.12.016 22498337

[35] Javed U Aftab W Ambrose JA Wessel RJ Mouanoutoua M Huang G Frequency of elevated troponin I and diagnosis of acute myocardial infarction. Am J Cardiol (2009) 104(1):9–13. 10.1016/j.amjcard.2009.03.003 19576313

[36] Melberg T Burman R Dickstein K. The impact of the 2007 ESC-ACC-AHA-WHF Universal definition on the incidence and classification of acute myocardial infarction: a retrospective cohort study. Int J Cardiol (2010) 139(3):228–33. 10.1016/j.ijcard.2008.10.021 19027971

[37] Morrow DA Wiviott SD White HD Nicolau JC Bramucci E Murphy SA Effect of the novel thienopyridine prasugrel compared with clopidogrel on spontaneous and procedural myocardial infarction in the trial to assess improvement in therapeutic outcomes by optimizing platelet inhibition with Prasugrel-Thrombolysis in myocardial infarction 38: an application of the classification system from the universal definition of myocardial infarction. Circulation (2009) 119(21):2758–64. 10.1161/CIRCULATIONAHA.108.833665 19451347

[38] Saaby L Poulsen TS Diederichsen AC Hosbond S Larsen TB Schmidt H Mortality rate in type 2 myocardial infarction: observations from an unselected hospital cohort. Am J Med (2014) 127(4):295–302. 10.1016/j.amjmed.2013.12.020 24457000

[39] Saaby L Poulsen TS Hosbond S Larsen TB Pyndt Diederichsen AC Hallas J Classification of myocardial infarction: frequency and features of type 2 myocardial infarction. Am J Med (2013) 126(9):789–97. 10.1016/j.amjmed.2013.02.029 23856021

[40] Stein GY Herscovici G Korenfeld R Matetzky S Gottlieb S Alon D Type-II myocardial infarction – patient characteristics, management and outcomes. PLoS One (2014) 9(1):e84285 10.1371/journal.pone.0084285 24392121 PMC3879301

[41] Muller KE Lavange LM Ramey SL Ramey CT. Power calculations for general linear multivariate models including repeated measures applications. J Am Stat Assoc (1992) 87(420):1209–26. 10.1080/01621459.1992.10476281 24790282 PMC4002049

[42] Surazynski A Palka J Wolczynski S. Acetylsalicylic acid-dependent inhibition of collagen biosynthesis and beta1-integrin signaling in cultured fibroblasts. Med Sci Monit (2004) 10(6):Br175–9. 15173663

[43] Demirtas S Karahan O Yazici S Guclu O Caliskan A Tezcan O Investigation of possible prophylactic, renoprotective, and cardioprotective effects of thromboprophylactic drugs against ischemia-reperfusion injury. Kaohsiung J Med Sci (2015) 31(3):115–22. 10.1016/j.kjms.2014.12.005 25744233 PMC11916766

[44] Szoka L Karna E Morka RP Palka JA. Enalapril stimulates collagen biosynthesis through prolidase-dependent mechanism in cultured fibroblasts. Naunyn Schmiedebergs Arch Pharmacol (2015) 388(6):677–83. 10.1007/s00210-015-1114-5 25772062 PMC4438220

[45] Zanaboni G Dyne KM Rossi A Monafo V Cetta G. Prolidase deficiency: biochemical study of erythrocyte and skin fibroblast prolidase activity in Italian patients. Haematologica (1994) 79(1):13–8. 15378943

[46] Liu G Nakayama K Awata S Tang S Kitaoka N Manabe M Prolidase isoenzymes in the rat: their organ distribution, developmental change and specific inhibitors. Pediatr Res (2007) 62(1):54–9. 10.1203/PDR.0b013e3180676d05 17515839

[47] Erbagci AB Araz M Erbagci A Tarakcioglu M Namiduru ES. Serum prolidase activity as a marker of osteoporosis in type 2 diabetes mellitus. Clin Biochem (2002) 35(4):263–8. 10.1016/S0009-9120(02)00305-3 12135686

[48] Altindag O Erel O Aksoy N Selek S Celik H Karaoglanoglu M. Increased oxidative stress and its relation with collagen metabolism in knee osteoarthritis. Rheumatol Int (2007) 27(4):339–44. 10.1007/s00296-006-0247-8 17096092

[49] Gejyo F Kishore BK Arakawa M. Prolidase and prolinase activities in the erythrocytes of patients with chronic uremia. Nephron (1983) 35(1):58–61. 10.1159/000183046 6888626

[50] Erkus E Altiparmak H Sezen H Kaya Z Gunebakmaz O Sezen Y Serum prolidase activity in patients with left ventricular diastolic dysfunction. Acta Cardiol (2015) 70(1):51–7. 10.1080/AC.70.1.3064593 26137803

[51] Demirbag R Yildiz A Gur M Yilmaz R Elci K Aksoy N. Serum prolidase activity in patients with hypertension and its relation with left ventricular hypertrophy. Clin Biochem (2007) 40(13–14):1020–5. 10.1016/j.clinbiochem.2007.05.015 17604013

[52] Bakuy V Gursoy M Hokenek F Gedikbasi A Atay M Nurdag A Prolidase activity in patients with coronary artery aneurysm. Angiology (2014) 65(7):574–9. 10.1177/0003319713491136 23748981

[53] Akcakoyun M Pala S Esen O Acar G Kargin R Emiroglu Y Dilatation of the ascending aorta is associated with low serum prolidase activity. Tohoku J Exp Med (2010) 220(4):273–7. 10.1620/tjem.220.273 20383038

[54] Suner A Nurdag A Polat M Kaya H Koroglu S Acar G Evaluation of serum prolidase activity in patients with slow coronary flow. Postepy Kardiol Interwencyjnej (2015) 11(3):206–11. 10.5114/pwki.2015.54015 26677361 PMC4631735

[55] Sezen Y Bas M Altiparmak H Yildiz A Buyukhatipoglu H Faruk Dag O Serum prolidase activity in idiopathic and ischemic cardiomyopathy patients. J Clin Lab Anal (2010) 24(4):213–8. 10.1002/jcla.20388 20626024 PMC6647631

[56] Hampson G Evans C Petitt RJ Evans WD Woodhead SJ Peters JR Bone mineral density, collagen type 1 alpha 1 genotypes and bone turnover in premenopausal women with diabetes mellitus. Diabetologia (1998) 41(11):1314–20. 10.1007/s001250051071 9833939

[57] Palka JA. The role of prolidase as an enzyme participating in the metabolism of collagen. Rocz Akad Med Bialymst (1996) 41(2):149–60. 9020526

[58] Leidig-Bruckner G Ziegler R. Diabetes mellitus a risk for osteoporosis? Exp Clin Endocrinol Diabetes (2001) 109(Suppl 2):S493–514. 10.1055/s-2001-18605 11460594

[59] Sayin R Aslan M Kucukoglu ME Luleci A Atmaca M Esen R Serum prolidase enzyme activity and oxidative stress levels in patients with diabetic neuropathy. Endocrine (2014) 47(1):146–51. 10.1007/s12020-013-0136-3 24347244

[60] Uzar E Tamam Y Evliyaoglu O Tuzcu A Beyaz C Acar A Serum prolidase activity and oxidative status in patients with diabetic neuropathy. Neurol Sci (2012) 33(4):875–80. 10.1007/s10072-011-0857-0 22120188

[61] Eren MA Torun AN Tabur S Ulas T Demir M Sabuncu T Serum prolidase activity in diabetic foot ulcers. Acta Diabetol (2013) 50(3):423–7. 10.1007/s00592-012-0448-4 23242638

[62] Sabuncu T Boduroglu O Eren MA Torun AN Aksoy N. The value of serum prolidase activity in progression of microalbuminuria in patients with type 2 diabetes mellitus. J Clin Lab Anal (2016) 30(5):557–62. 10.1002/jcla.21902 26666214 PMC6807120

[63] Li W Li M Gao C Wang X Qi D Liu J Impact of type 2 diabetes mellitus on recurrent myocardial infarction in China. Diab Vasc Dis Res (2016) 13(6):395–404. 10.1177/1479164116653606 27390227

[64] Mondesir FL Brown TM Muntner P Durant RW Carson AP Safford MM Diabetes, diabetes severity, and coronary heart disease risk equivalence: reasons for geographic and racial differences in stroke (REGARDS). Am Heart J (2016) 181:43–51. 10.1016/j.ahj.2016.08.002 27823692 PMC5117821

[65] Andersson J Wennberg P Lundblad D Escher SA Jansson JH. Diabetes mellitus, high BMI and low education level predict sudden cardiac death within 24 hours of incident myocardial infarction. Eur J Prev Cardiol (2016) 23(17):1814–20. 10.1177/2047487316659574 27435083

[66] Scirica BM Bhatt DL Braunwald E Raz I Cavender MA Im K Prognostic implications of biomarker assessments in patients with type 2 diabetes at high cardiovascular risk: a secondary analysis of a randomized clinical trial. JAMA Cardiol (2016) 1(9):989–98. 10.1001/jamacardio.2016.3030 27681000

[67] Tailakh MA Friger M Zahger D Sidi A Mazor-Dray E Novack V Prospective study of the impact of diabetes mellitus newly diagnosed by glycated hemoglobin on outcomes in patients undergoing percutaneous coronary intervention. Eur J Intern Med (2017) 37:69–74. 10.1016/j.ejim.2016.09.007 27665509

